# HPLC Analysis, Optimization of Extraction Conditions and Biological Evaluation of *Corylopsis coreana* Uyeki Flos

**DOI:** 10.3390/molecules21010094

**Published:** 2016-01-15

**Authors:** Ji-Hye Seo, Jung-Eun Kim, Jung-Hyun Shim, Goo Yoon, Mi-Ae Bang, Chun-Sik Bae, Kyung-Jin Lee, Dae-Hun Park, Seung-Sik Cho

**Affiliations:** 1 Department of Oriental Medicine Materials, Dongshin University, Naju, Jeonnam 520-714, Korea; wlgpsid7156@naver.com; 2 Department of Pharmacy, College of Pharmacy, Mokpo National University, Muan, Jeonnam 534-729, Korea; wjddms1568@nate.com (J.-E.K.); s1004jh@gmail.com (J.-H.S.); gyoon@mokpo.ac.kr (G.Y.); 3 Research Develpoment Team, Jeonnam Bioindustry Foundation, Food Research Institute, Naju, Jeonnam 520-330, Korea; methyl@nate.com; 4 College of Veterinary Medicine, Chonnam National University, Gwangju 500-757, Korea; csbae210@chonnam.ac.kr; 5 Department of Convergence Medicine, Asan Institute for Life Sciences, University of Ulsan College of Medicine, Asan Medical Center, Seoul 05505, Korea; kjlee@amc.seoul.kr

**Keywords:** *Corylopsis coreana* Uyeki flos, simultaneous analysis, HPLC, flavonoid, isocoumarin

## Abstract

A method for the separation and quantification of three flavonoids and one isocoumarin by reverse-phase high performance liquid chromatography (HPLC) has been developed and validated. Four constituents present in a crude ethanolic extract of the flowers of *Coryloposis coreana* Uyeki, were analyzed. Bergenin, quercetin, quercitrin and isosalipurposide were used as calibration standards. In the present study, an excellent linearity was obtained with an *r*^2^ higher than 0.999. The chromatographic peaks showed good resolution. In combination with other validation data, including precision, specificity, and accuracy, this method demonstrated good reliability and sensitivity, and can be conveniently used for the quantification of bergenin, quercetin, quercitrin and isosalipurposide in the crude ethanolic extract of *C. coreana* Uyeki flos. Furthermore, the plant extracts were analyzed with HPLC to determine the four constituents and compositional differences in the extracts obtained under different extraction conditions. Several extracts of them which was dependent on the ethanol percentage of solvent were also analyzed for their antimicrobial and antioxidant activities. One hundred % ethanolic extract from *C. coreana* Uyeki flos showed the best antimicrobial activity against the methicillin-resistant *Staphylococcus aureus* (MRSA) strain. Eighty % ethanolic extract showed the best antioxidant activity and phenolic content. Taken of all, these results suggest that the flower of *C. coreana* Uyeki flos may be a useful source for the cure and/or prevention of septic arthritis, and the validated method was useful for the quality control of *C. coreana* Uyeki.

## 1. Introduction

*Corylopsis coreana* Uyeki belongs to the family Hamamelidaceae and is cultivated in South Korea as an ornamental plant. There are few research reports about species of the genus *Corylopsis*, such as *C. coreana* Uyeki. In a previous report, fifteen compounds from the leaves of *C. coreana* Uyeki were identified and their anti-oxidative and anti-proliferative properties were evaluated [1].

On the other hand, *Hamamelis virginiana* (witch hazel) which has been used as a culinary materials in North America has been widely investigated. *H*. *virginiana* bark is a source for both proanthocyanidins, or condensed tannins, and hydrolyzable tannins. Witch hazel has been used as a traditional medicine in uses such the treatment of hemorrhages, skin irritation, and inflammatory disease [2,3,4] and it was recently reported that hamamelitannin from the bark of witch hazel has anti-cancer activity [5]. On the other hands, there are no reports about analysis and/or biological properties of extracts from *Corylopsis coreana* Uyeki flos. So herein we investigated active constituents and the biological activities of extracts from *C. coreana* Uyeki flos for the development of possible alternatives to *H. virginiana*.

In a previous report, quercetin, quercitrin and bergenin were identified from the leaves of *C. coreana* Uyeki [6]. In present study we identified quercetin, quercitrin and bergenin from the flower of *C. coreana* Uyeki and identified isosalipurposide for the first time.

Bergenin, quercetin and quercitrin have been shown to play important roles in inflammation and microbial infection. Quercetin is a natural flavonoid with various pharmacological effects, such as improving flow mediated dilation,lowering blood pressure, and anti-inflammatory, anti-allergy, anti-platelet aggregation, antimicrobial and antitumor effects [7,8]. It is present in many fruits, vegetables, seeds, barks and leaves and mainly exists in a glycosylated form such as quercitrin. Quercitrin is a bioflavonoid with antioxidant properties and is better absorbed than other forms of quercetin. Quercitrin also has various actions such as protecting the skin from UV damage, and anti-microbial, antitumor and anti-allergy effects [9]. Bergenin is a natural isocoumarin that has been reported to have anti oxidant, antibacterial, inhibiting cholinesterase activity, neuroprotective, anti-inflammatory, gastroprotective, hypolipidaemic, anti-HIV, anti arrhythmic, PTP1B inhibitory, and anti-fungal activities [10,11]. Isosalipurposide is a natural dihydrochalcone with a structure similar to phlorizin. More than 700 articles dealing with phlorizin or its derivatives have been published since 2000. Most studies relate to diabetes, obesity, stress, hyperglycemia and antioxidant activity [12]. However, it has only been reported that isosalipurposide has been isolated from *Nympharea caerulea* flos and *Helicgrysu maracandicum* with antioxidant and anticarcinogenic activities being reported [13,14]. We recently reported cytoprotective effects against oxidative injury of hepatocytes [15].

Standard profile for quality control in natural products utilization is one of important steps. Chemical and chromatographic techniques may be used to aid the identification of herbal extract [16]. However there is no standard profile for *C. coreana* Uyeki flos so in this study we established the quality control method using with HPLC which could separate and quantify bergenin, quercetin, quercitrin, and isosalipurpoide from ethanolic extract of *C. coreana* Uyeki flos. There is currently no other validated HPLC method reported for the simultaneous determination of bergenin, quercetin, quercitrin and isosalipurposide in the Hamamelidaceae species.

## 2. Results and Discussion

### 2.1. Optimization of the Chromatographic Conditions

**Figure 1:**
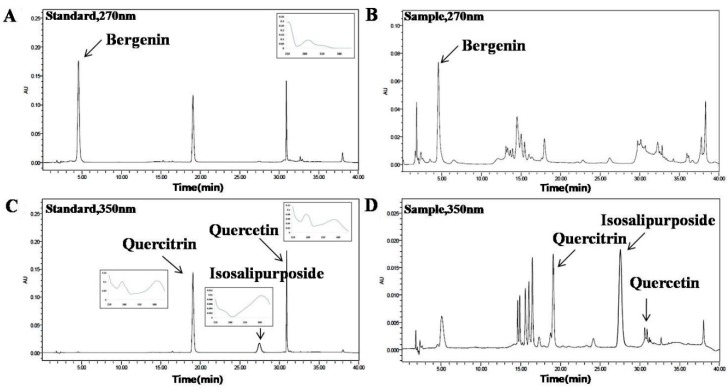
Identification of active compounds in *C. coreana* Uyeki flos by HPLC. (**A**) standard mixture at 270 nm; (**B**) sample extract at 270 nm; (**C**) standard mixture at 350 nm; (**D**) sample extract at 350 nm, A mobile phase consisting of mixture of solvent A (acetonitrile) and B (water containing 0.2% phosphoric acid) and employing a gradient elution (from 10:90 to 100:0, *v*/*v*) at a flow rate of 0.8 mL/min. The detection wavelength was set at 270 nm for bergenin, at 350 nm for quercetin, quercitrin and isosalipurposide.

The ratio of methanol and water containing phosphoric acid as the mobile phase, column temperature, wavelength and flow rate were investigated for good separation (data not shown). A gradient program was used to separate the four active markers in a single run within a reasonable period of time. In the present study, two different detection wavelengths were set according to the UV absorption maxima of the compounds. Bergenin was detected at 270 nm, quercetin, quercitrin and isosalipurposide were detected at 350 nm. Under the proposed analytical conditions, baseline resolution was obtained for all the analytes. Chromatograms of the standards and sample solutions are shown in Figure 1.

### 2.2. Method Validation

#### 2.2.1. System Suitability

For method validation process, System suitability test were examined. The result shows that system suitability indicates 0.127%–0.5% RSD 0.25–0.43, plate number 2061–169744, tailing factor 0.83–1, and resolution 2.5–91.7. All results were obtained in acceptable ranges (Table 1).

**Table 1:** System suitability testing of four marker compounds.

Compound	Theoretical Plate Number (N)	Tailing Factor (T)	Resolution (Rs)	Repeatability of Retention Time (RSD %)	Conclusion
Accepted criteria	N > 2000	T ≤ 2.0	R ≥ 1.5	RSD% < 1.0	Satisfaction
Bergenin	2061	1.00	2.5	0.43	Satisfaction
Quercitrin	23,250.2	0.95	39.2	0.3	Satisfaction
Isosalipuropside	169,744.0	0.83	91.7	0.36	Satisfaction
Quercetin	27676.9	1.00	50.1	0.25	Satisfaction

#### 2.2.2. Limit of Detection (LOD) and Limit of Quantification (LOQ)

The LOD of an individual analytical procedure is the lowest amount of an analyte in a sample that can be detected but not necessarily quantified. The LOQ of an individual analytical procedure is the lowest amount of analyte in a sample that can be determined with suitable precision and accuracy. The LOD was found to be 1.79, 0.07, 0.07 and 0.32 μg/mL for bergenin, quercetin, quercitrin and isosalipurposide, respectively. The LOQ values for bergenin, quercetin, quercitrin and isosalipurposide, were found to be 5.92, 0.23, 0.24 and 1.06 μg/mL, respectively (Table 2).

#### 2.2.3. Linearity

Calibration curves were linear over a large concentration range of 6.25–100 μg/mL for bergenin, 3.125–50 μg/mL for quercetin/quercitrin and 12.5–200 μg/mL for isosalipurposide. Calibration curves exhibited good linear regressions (*r*^2^ = 0.9999 for bergenin, *r*^2^ = 0.9999 for quercitrin, *r*^2^ = 0.9999 for isosalipurposide and *r*^2^ = 0.9999 for quercetin) (Table 2).

**Table 2:** HPLC data for the calibration graphs and limit of quantification of the four active compounds.

Analyte	Retention Time (min)	*R*^2^	Linear Range (μg/mL)	LOQ (μg/mL)	LOD (μg/mL)
Bergenin	4.6	0.9999	6.25–100	5.92	1.79
Quercitrin	19.1	0.9999	3.125–50	0.24	0.07
Isosalpurposide	27.5	0.9999	12.5–200	1.06	0.32
Quercetin	30.1	0.9999	3.125–50	0.23	0.07

#### 2.2.4. Precision and Accuracy

The results of the intra-day and inter-day precision experiments are shown in Table 2. The developed method was found to be precise as the Relative Standard Deviation (RSD) values for repeatability of intra-day and inter-day precision studies were below 2.5%, which is under the limit as per recommendations of the International Conference for Harmonisation (ICH) guidelines [17]. The overall recovery percentages were in the range of 97.31%–102.56% for bergenin, 98.94%–102.47% for quercitrin, 97.83%–101.16% for quercetin and 98.23%–101.99% for isosalipurposide. These results demonstrate that the developed method is reproducible with a good accuracy (Table 3).

**Table 3:** Analytical results of intra-day and inter-day precision and accuracy.

Analyte	Conc (μg/mL)	Intra-Day (*n* = 3)		Inter-Day (*n* = 3)	
		RSD (%) ^a^	Accuracy (%)	RSD (%)	Accuracy (%)
Bergenin	12.5	1.35	102.56	1.66	102.51
	25	1.05	98.470	0.12	98.54
	50	0.44	97.31	2.40	99.98
Quercitrin	6.25	1.06	97.49	1.57	98.77
	12.5	0.66	97.83	0.84	98.79
	25	0.60	99.03	2.23	101.16
Isosalpurposide	25	0.86	98.23	1.77	99.47
	50	0.63	98.81	0.40	99.39
	100	0.55	100.08	2.28	101.99
Quercetin	6.25	0.78	98.94	1.52	100.22
	12.5	0.82	99.20	1.03	100.02
	25	0.72	100.24	2.28	102.47

**^a^** RSD: relative standard deviation.

#### 2.2.5. Repeatability

The results of the repeatability experiments are shown in Table 4. The developed method was found to be precise as the RSD values for the repeatability precision studies were below 1.0%.

**Table 4:** Analytical data of recovery (*n* = 6).

Analyte	Added (μg/mL)	Recovery (%) (Mean ± SD)	RSD (%) ^a^
Bergenin	12.5	100.77 ± 0.50	0.55
	25	98.32 ± 0.62	0.67
	50	98.64 ± 0.37	0.38
Quercitrin	6.25	98.37 ± 0.68	0.69
	12.5	99.44 ± 0.96	0.96
	25	99.50 ± 0.75	0.75
Isosalpurposide	25	98.75 ± 0.68	0.68
	50	99.01 ± 0.99	1.00
	100	99.87 ± 0.77	0.77
Quercetin	6.25	98.43 ± 0.85	0.86
	12.5	99.75 ± 1.02	1.02
	25	99.67 ± 0.85	0.85

**^a^** RSD: relative standard deviation.

### 2.3 Antimicrobial Activity of C. coreana Uyeki Flos Extracts

**Figure 2:**
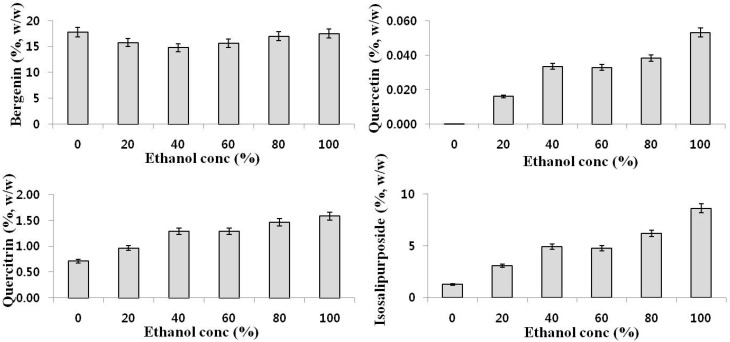
Concentration of bergenin, quercetin, quercitrin and isosalipurposide in ethanolic extract from *C. coreana* Uyeki flos. Each value was the mean ± SD (*n* = 3).

Samples were extracted with six different solvent compositions in order to select the best extraction solvent conditions: water, 20% ethanol, 40% ethanol, 60% ethanol, 80% ethanol and 100% ethanol (*v*/*v*). Firstly, we analyzedthe inhibition effects of the extract from *C. coreana* Uyeki flos on methicillin resistant *Staphylococcus aureus* 693E (MRSA 693E) through the disk diffusion method and found that extract displayed antimicrobial activity (Table 5). The 100% ethanolic extract showed the best antimicrobial activity. In Table 4, quercetin was used as control. Quercetin showed antimicrobial activity against MRSA 693E with 400 μg of quercetin loading on the disc. Accordinh to Figure 2, the ethanolic extract contains very low levels of quercetin. After purifying unknown antimicrobial compounds, which is now in progress, we will study the antimicrobial effect of the extract in detail in relation to their corresponding reference compounds. Secondly, a HPLC method was applied to analyze the six samples. The average amounts (%wt) of bergenin, quercetin, quercitrin and isosalipurposide are presented in Figure 2. The amount of the four compounds in the 100% ethanolic extract was higher than that of in the 0%–80% ethanolic extracts. According to these results, a 100% ethanol solution was selected as the most effective extraction solvent.

**Table 5:** Antimicrobial activity of extract from *C. coreana* Uyeki flos.

Ethanolic Extract (Ethanol %, *v*/*v*)	Zone of Inhibition ± SD (cm, Diameter of disc:0.8)
Vancomycin (10 μg/disc)	1.3 ± 0.1
Quercetin (400 μg/disc)	1.07 ± 0.06
0	0.8
20	0.8
40	0.93 ± 0.06
60	1.07 ± 0.06
80	1.20 ± 0.1
100	1.23 ± 0.06

Each values was the mean ± SD (*n* = 3).

### 2.4. Antioxidant Activity and Total Phenolic Contents of C. coreana Uyeki Flos Extracts

The antioxidant potential of various extracts of *C. coreana* Uyeki flos was determined by the 2,2-diphenyl-1-picrylhydrazyl (DPPH) and reducing power assays. Plants have been reported to be plentiful sources of phytochemicals such as phenolics and various health benefits like antioxidant activity have been suggested [18]. In addition, Cho *et al.* [19] reported that the various antioxidant activities of natural resources were significantly correlated with their major compound contents, such as polyphenols. Therefore, we determined the total phenolic contents of extracts obtained from *C. coreana* Uyeki flos.

The DPPH scavenging effect is a widely used method to evaluate the free radical scavenging ability of different samples, including plant extracts. The measured DPPH radical scavenging activity is shown in Table 5. A low IC_50_ value indicates strong antioxidant activity in a sample. The scavenging effect from IC_50_ data on the DPPH radical increased in the order: water extract (182.3 ± 0.004 µg/mL) > 20% ethanol extract (139.3 ± 0.005 µg/mL) > 40% ethanol extract (100 ± 0.07 µg/mL) > 60% ethanol extract (90 ± 0.001 µg/mL) > 100% ethanol extract (56.1 ± 0.004 µg/mL) > 80% ethanol extract (30.6 ± 0.01 µg/mL).

Fe^3+^ was transformed into Fe^2+^ in the presence of extracts to measure the reductive capability. Eighty % etanolic extract had highest activity among the six extracts. At 100 µg/mL, the values, which were expressed as vitamin C equivalents, decreased in the order: 80% ethanol extract (64.33 ± 5.05 µg/mL) > 60% ethanol extract (62.93 ± 3.56 µg/mL) > 40% ethanol extract (62.63 ± 1.39 µg/mL) > 20% ethanol extract (42.21 ± 2.53 µg/mL) > water extract (37.38 ± 1.26 µg/mL) > 100% ethanol extract (25.58 ± 6.65 µg/mL).

Total phenol compounds, as determined by the Folin Ciocalteu method [20], is reported as gallic acid equivalents by reference to standard curve (*r*^2^ = 0.999). The total phenolic content of extracts is shown in Table 6. The amount of the phenolic compounds in the 80% ethanolic extract was higher than that of in the 0%–60% and 100% ethanolic extracts. Eighty percentage ethanolic extract showed the best DPPH radical scavenging activity, reducing power and phenolic contents. Antioxidant and phenolic contents of 100% ethanolic extract was less than 80% ethanolic extract.

**Table 6:** Antioxidant activity and total phenolic contents of extracts of *C. coreana* flos.

Extract	DPPH Assay (IC_50_, μg/mL)	Reducing Power (Ascorbic Acid eq. μg/100 μg Extract)	Total Phenolic Content (Gallic Acid eq. mg/g)
Ascorbic acid	4.25 ± 0.04		
Water	182.3 ± 0.004	37.88 ± 1.26	111.47 ± 5.1
20% EtOH Ex	139.3 ± 0.005	41.21 ± 2.53	138.13 ± 17.87
40% EtOH Ex	100 ± 0.07	62.63 ± 1.39	220 ± 14.7
60% EtOH Ex	90 ± 0.001	62.93 ± 3.56	245.07 ± 23.17
80% EtOH Ex	30.6 ± 0.01	64.33 ± 5.05	269.37 ± 98.72
100% EtOH Ex	56.1 ± 0.01	25.58 ± 6.65	140.53 ± 14.95

Kim, *et al* reported that content of caffeic acid derivatives from *Ligularia fischeri* increased when extracted with hot water (116 μg/mL). But 50% and 100% ethanolic extracts, contents of caffeic acid derivatives were 72 and 11 μg/mL [21]. Similarly, we previously reported that extraction efficiency of caffeic acid from pear pomace decreased with increasing percentage of ethanol. Besides, the extraction efficiency of chlorogenic acid from pear pomace increased with increasing amount of ethanol [22]. We concluded that phenolic extraction maybe affected by solvent combinations. In our case, 80% ethanol was a more efficient solvent in the extraction of phenolic compounds from *C. coreana*.

From the ethanolic extracts from *C. coreana* flos, we identified phenolic compounds such as bergenin, quercetin, quercitrin. They are phenolic compounds widely found in food products derived from plant sources, and they have been shown to possess antioxidant activities [23,24,25].

### 2.5. C. coreana Uyeki Flos: A Potential Natural Source for the Treatment of Infectious Arthritis

Septic arthritis is an acute or chronic infectious disease that mainly occurs in a native or prosthetic joint. Timely and appropriate treatment can effectively reduce morbidity [26]. American studies estimated an occurrence of 20,000 cases of suppurative arthritis per year (7.8 individuals per 100,000/year). In Denmark from 2003 to 2004 *Streptococcal* infections occurred at a rate of 2.6 cases per 100,000 people [27]. To date, interest in the use of antibiotics in arthritis treatment has been motivated by two factors: (1) in some forms of chronic arthritis, microbial antigens persist in the synovial membrane; and (2) the increasing knowledge of the anti-inflammatory and immunosuppressive effects of antibiotics. Several studies have reported a beneficial effect of antibiotics such as tetracycline and ciprofloxacin on rheumatoid arthritis and reactive arthritis [28,29]. The adverse effects seem to be mild, however the long-term efficacy and safety of tetracycline as a disease-modifying anti rheumatic drug remain to be demonstrated. In particular the eradication of methicillin resistant *Staphylococcus aureus* (MRSA) that can produce extended-spectrum β-lactamases (ESBL) and carbapenemases is urgent [30,31].

Recently there has been great effort to find candidates from natural products to effectively control infectious strains. For instance, flower extracts of *Calotropis procera* [32] and *Delonix regia* [33] have been reported to exhibit antimicrobial activity against infectious strains. Extracts of various plants containing flavonoids and isocoumarins have also been previously reported to possess antimicrobial activity. The flavonoids quercetin and quercitrin have antimicrobial activities against general infectious bacteria such as *Staphylococcus aureus*, *Staphylococcus epidemidis*, *Streptococcus pyrogenes* and *Pseudomonas* *aeruginosa* [34]. The isocoumarin bergenin also has antimicrobial activities against the *Candida* species and some *Aspergillus* species [35]. Recent reports show that phenolic compounds have curative effects on septic arthritis. For example, chlorogenic acid is known as one of the most abundant polyphenols in the human diet. It has activity against arthritis caused by *Candida albicans* [36]. Gentamicin in combination with ascorbic acid regulates the severity of *Staphylococcus aureus* infection-induced septic arthritis in mice [37].

From the HPLC analysis, we found that the amount of bergenin was the highest among the four identified compounds in the extract from *C. coreana* Uyeki flos, with isosalipurposide being the second. Quercitrin and quercetin were minor compounds in the extract. Based on the analytical data, we focused on the anti-microbial, antioxidant and anti-inflammatory activities of the two flavonoids and one isocoumarin but not isosalipurposide.

First, we focused on bergenin. One hundred percent ethanolic extract of *C. coreana* Uyeki flos contained bergenin with a concentration of 17.5% (*w*/*w*). Luis *et al.* found that contents of bergenin were 15.7% and 20% (*w*/*w*) in 50% ethanolic and hot water extract from *Endopleura uchi* [11]. Our result indicate that extracts *C. coreana* Uyeki flos are a rich source of bergenin similar to the extract from *Endopleura uchi* and there are no prior reports about the presence of bergenin in *C. coreana* Uyeki flos.

Bergenin was reported to have anti-inflammatory activity [38,39]. Additionally, bergenin showed antinociceptive properties in models of inflammatory pain and did not show any apparent systemic toxicity [31]. Thus, bergenin may be a useful compound for the treatment of arthritis. We suggest that bergenin and extracts containing bergenin may be good candidates for the treatment of arthritis caused by microbial infection.

Secondly we considered quercetin and quercitrin. Guardia, *et al.* found that quercetin has anti-inflammatory effects in rat adjuvant arthritis [40] and can reduce the production of macrophage inflammatory mediators in the adjuvant-induced arthritis mouse model [41]. Quercetin has minor side effects. In clinical study phase I, the recommended dose of quercetin is 1400 mg/m^2^, which corresponds to approximately 2.5 g for a 70 kg individual [42]. For a 4 g single-dose oral administration or 500 mg twice daily, no side effects were found in through the repeated dosing study [43]. In the present study, quercetin and quercitrin where found in a ratio of 1:32 in the 100% ethanolic extract of *C. coreana* Uyeki flos. The extract shows antimicrobial activity and contains four pharmacologically active compounds. We examined the pharmacological activities of two flavonoids (quercetin and quercitrin) and an isocoumarin (bergenin) and the mild side effects. We conclude that the validation method for *C. coreana* Uyeki flos is quite valuable and this plant is a promising antimicrobial and anti-inflammatory drug source candidate.

## 3. Experimental Section

### 3.1. Plant Material and Preparation of the Extract

*C. corean* Uyeki flos was collected in May 2013 near the Jogye Mountain, in Jeonnam Province, Korea. A voucher specimen (MNUCSS-CC-01) was deposited in the College of Pharmacy, Mokpo National University. Only the flowers were separated for the present study. The air-dried, powdered *C. coreana* Uyeki flos (10 g) was extracted twice with 80% ethanol (100 mL) at room temperature for 3 days. After filtration the ethanol was evaporated, freeze-dried and stored at −50 °C. Crude extract was resuspended in ethanol and filtered using a 0.4 μm membrane.

### 3.2. Instrumentation and Chromatographic Conditions

All analysis was performed on an Alliance 2695 HPLC system (Waters, Millford, MA, USA) equipped with a photodiode array detector. The analytical column used was an Agilent Zorbax extended C18 (5 µm, 150 mm × 5 mm) with a mobile phase consisting of mixture of solvent A (acetonitrile) and B (water containing 0.2% phosphoric acid) and employing gradient elution (from 10/90 to 100/0, *v*/*v*) at a flow rate of 0.8 mL/min (Table 7). The column temperature was maintained at 25 °C and the detection wavelength was set at 270 nm for bergenin, at 350 nm for quercetin, quercitrin and isosalipurposide. The solvent was filtered through a 0.22 µm filter and degassed. The sample injection volume was 10 µL.

**Table 7:** Analytical conditions of HPLC for analysis of the four standards.

Parameters	Conditions		
Column	Zorbax extended-C18 (C18, 4.6 mm × 150 mm, 5 µm)		
Flow rate	0.8 mL/min		
Injection volumn	10 μL		
UV detection	270 nm, 350 nm		
Run time	40 min		
Gradient	**Time (min)**	**% A** ^1^	**% B** ^2^
	0	10	90
	10	10	90
	11	20	80
	27	20	80
	30	100	0
	35	100	0
	36	10	90
	40	10	90

^1^ Acetonitrile; ^2^ 0.2% phosphoric acid.

### 3.3. Preparation of Standards and Sample Solutions

#### 3.3.1. Standard Solutions

Accurately weighed appropriate amounts of the reference compounds (bergenin, quercetin, quercitrin and isosalipurposide) were mixed and dissolved in methanol in a 100 mL volumetric flask, to obtain a stock solution of 200 μg/mL. Solutions were subsequently 2-fold serially diluted to 3.125 μg/mL.

#### 3.3.2. Sample Solutions

Crude extract (0.5 g) was dissolved in methanol (10 mL). This sample was sonicated to speed up the dissolution of particles. Subsequently, 1 mL was transferred to a volumetric flask and diluted in 9 mL of mobile phase A. A final solution with a known concentration of 5 mg/mL was thus obtained.

### 3.4. Method Validation

In the validation of the analytical method used for the quantification of bergenin, quercetin, quercitrin and isosalipurposide in the ethanolic extract of *C. coreana* Uyeki flos, the following parameters were determined: specificity, linearity, sensitivity, accuracy, precision and recovery. Before starting the analysis, system suitability testing was performed [17].

#### 3.4.1. Specificity

Specificity is the ability of a method to discriminate between the study analytes and other constituents in the sample. Specificity of the HPLC method is demonstrated by the separation of the analytes from other potential constituents such as impurities, degradants, or excipients [44]. The resolution between the peaks of the main constituents found in the ethanolic extract of *C. coreana* Uyeki flos was determined by the analysis of chromatograms of the standard solution and the sample solution. This resolution was calculated using the Waters Empower software (version 1).

#### 3.4.2. Linearity

The linearity was analyzed using three calibration curves obtained with standard solutions at five different concentrations from the following concentration ranges; 6.25–100 μg/mL for bergenin; 3.12–50 μg/mL for quercetin and quercitrin; and 12.5–200 μg/mL for isosalipurposide. The data for the peak area *vs.* the drug concentration were treated with linear regression analysis using Excel^®^ software.

#### 3.4.3. Sensitivity

The limit of detection (LOD) and the limit of quantification (LOQ) were determined from the calibration curves of the bergenin, quercetin, quercitrin and isosalipurposide standards. The LOD was calculated according to the expression SDR × 3/S, where SDR is the standard deviation of the response and S is the slope of the calibration curve. The LOQ was established using the expression SDR × 10/S.

#### 3.4.4. Accuracy and Precision

The accuracy and precision were evaluated by means of recovery assays carried out by adding known amounts of the standards to the sample, at three different levels (12.5, 25 and 50 μg/mL for bergenin; 6.25, 12.5 and 25 μg/mL for quercetin and quercitrin; 25, 50 and 100 μg/L for isosalipurposide) of the initial concentration of the sample. Each solution was injected in triplicate within 1 day or 3 sequential days. Intra- and inter-day accuracy was expressed as the observed concentration value relative to the true concentration value. Intra- and inter-day precisions were expressed as the relative standard deviation (RSD).

#### 3.4.5. Recovery

Recovery was accessed by analyzing the peak areas using six determinations at three different levels (12.5, 25 and 50 μg/mL for bergenin; 6.25, 12.5 and 25 μg/mL for quercetin and quercitrin; 25, 50 and 100 μg/mL for isosalipurposide). Variations were expressed as % of the standard concentration and the relative standard deviation (RSD).

#### 3.4.6. Statistical Analysis

The data were subjected to statistical analysis using Excel^®^ software.

### 3.5. Analysis of the Extract from C. coreana Uyeki Flos

The HPLC method developed in the present study was used to quantitatively determinate the amounts of three flavonoids and one isocoumarin contents in 5 extracts from *C. coreana* Uyeki flos.

### 3.6. Antimicrobial Assay

The standard plate assay technique described by Iwasa *et al.* [45] with some modifications was employed to determine the effect of *C. coreana* Uyeki flos on MRSA 693E. *C. coreana* Uyeki flos extracts (1 mg/40 μL), quercetin (400 μg/disc) and vancomycin (10 μg/disc) were loaded onto paper discs (sterilized, Adventec, Toyo, Kaisha Ltd., Tokyo, Japan) then transferred to Mueller-Hinton agar plate containing MRSA 693E of 10^6^ colony forming units (CFU). In this experiment, ethanol did not inhibit MRSA 693E growth (data not shown). Quercetin and vancomycin were selected to quantitatively compare the antimicrobial activity between internal/external markers and extracts. The plate was incubated at 37 °C and the clear inhibition zone that appeared surrounding the paper disc was measured using a digital caliper after 24 h.

### 3.7. DPPH Free Radical Assay

Antioxidant activity determination of the different extracts was performed by the DPPH radical scavenging method. DPPH radicals have an absorption maximum of 517 nm, which disappears with reduction by an antioxidant compound. ethanolic solution (1 mL) containing 1 to 20 mg of extract was added to a 0.4 mM DPPH ethanolic solution (1 mL). The solution was mixed and allowed to react at room temperature in the dark for 10 min. The absorbance at 517 nm was measured using a microplate reader(Perkin Elmer, Waltham, MA, USA). The radical scavenging activity was calculated as a percentage using the following equation:

DPPH radical scavenging activity (%) = [1 − (Asample/Ablank)] × 100

DPPH free radical scavenging activities of samples compared in terms of their IC_50_ (μg/mL) values [20].

### 3.8. Reducing Power

The reducing power of the sample was determined according to a modified reducing power assay method. The sample (0.1 mL) was added to 0.2 M sodium phosphate buffer (0.5 mL) and 1% potassium ferricyanide (0.5 mL), and this mixture was incubated at 50 °C for 20 min. Following incubation, 10% trichloroacetic acid solution (0.5 mL) was added to the reaction mixture, and it was centrifuged at 12,000 rpm for 10 min. The supernatant was mixed with distilled water (0.5 mL) and a 0.1% iron (III) chloride solution (0.1 mL), and the absorbance at 700 nm of the resulting solution was measured. Reducing powers of samples were expressed as vitamin C equivalents [20]. 

### 3.9. Determination of Total Phenolic Content

The total phenolic content was determined using Folin-Ciocalteu assay. Water solution (1 mL) containing 5 mg of the freeze-dried extract or standard was mixed with 1 mL of 2% sodium carbonate solution and 1 mL of 10% Folin-Ciocalteu’s phenol reagent. After 10 min, the absorbance was measured at 750 nm using a microplate reader (Perkin Elmer). The measurement was compared to calibration curve of gallic acid. The results were expressed as milligrams of gallic acid equivalents per gram of sample [20].

## 4. Conclusions

It was demonstrated here for the first time that three flavonoids and one isocoumarin were present in *C. coreana* Uyeki flos extract and it could be confirmed that bergenin is the major compound in the flowers, as described in the literature. High-performance liquid chromatography has been used to quantify active compounds in *C. coreana* Uyeki flos extracts. One hundred % ethanolic extract of *C. coreana* Uyeki flos contained bergenin with a concentration of 17.5% (*w*/*w*). The isocoumarin was identified as isosalipurposide (8.64% *w*/*w*). Quercetin and quercitrin were found in the extract of *C. coreana* Uyeki flos in a ratio of 1:32 (0.05%, 1.6% *w*/*w*). The inhibition effect of the extract on a methicillin resistant *Staphylococcus aureus* (MRSA) strain was analyzed by the disc diffusion method. One hundred % ethanolic extract of *C. coreana* Uyeki flos showed the best antimicrobial activity. Furthermore, 80% ethanolic extract showed the best antioxidant activity and phenolic content. These findings led us to suggest that the specific antimicrobial, antioxidant anti inflammatory effects of *C. coreana* Uyeki flos extract could be attributed, at least in part, to the presence of bergenin, isosalipurposide, quercetin and quercitrin. Further investigations are needed to confirm the pharmacological activity of the four compounds present in *C. coreana* Uyeki flos extract in animal models, and to assess the safe use of the plant, which could lead to its potential development as an effective antimicrobial, antioxidant and anti inflammatory agent.
